# In Well‐Treated Celiac Patients Low‐Level Mucosal Inflammation Predicts Response to 14‐day Gluten Challenge

**DOI:** 10.1002/advs.202003526

**Published:** 2021-01-04

**Authors:** Jorunn Stamnaes, Daniel Stray, Maria Stensland, Vikas K. Sarna, Tuula A. Nyman, Knut E. A. Lundin, Ludvig M. Sollid

**Affiliations:** ^1^ K.G. Jebsen Coeliac Disease Research Centre University of Oslo Oslo Norway; ^2^ Department of Immunology University of Oslo and Oslo University Hospital‐Rikshospitalet Oslo 0372 Norway; ^3^ Department of Gastroenterology Oslo University Hospital‐Ullevål Oslo 0450 Norway; ^4^ Department of Gastroenterology Oslo University Hospital‐Rikshospitalet Oslo 0372 Norway

**Keywords:** celiac disease, gluten challenge, laser capture microdissection, mass spectrometry, tissue proteomics

## Abstract

In celiac disease (CeD), gluten activates adaptive immune cells that cause damage to the small intestinal mucosa. Histological evaluation of intestinal biopsies allows for grading of disease severity. CeD can effectively be treated with a life‐long gluten‐free diet. Gluten challenge of treated CeD patients is used to confirm diagnosis and to test drug efficacy in clinical trials, but patients respond with different magnitudes to the same gluten challenge. In this study of 19 well‐treated CeD patients, proteome analysis of total tissue or isolated epithelial cell compartment from formalin‐fixed paraffin embedded biopsies collected before and after 14‐day gluten challenge demonstrates that patients with strong mucosal response to challenge have signs of ongoing tissue inflammation already before challenge. This low‐level tissue inflammation at baseline is paralleled by increased gluten specific CD4+ T‐cell frequencies in the gut and presence of a low‐level blood inflammatory profile. Thus, apparently well‐treated CeD is frequently not entirely quiescent, with presence of low‐grade inflammation and antigluten immunity in the gut mucosa. Histology assessment alone appears insufficient to judge full recovery and gut mucosal healing of CeD patients. The findings raise a concern whether a seemingly proper gluten‐free diet is able to curb gut inflammation in all CeD patients.

## Graphical abstract

Spatially resolved tissue proteome analysis reveals low‐level inflammation and antigluten immunity in gut of some patients that are apparently well‐treated with gluten‐free diet. These patients develop strong mucosal response to short‐term gluten challenge.

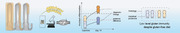

Celiac disease (CeD) is a prevalent disorder that severely affects the upper part of the small intestine and impairs health and quality of life of many children and adults. The disease occurs in genetically susceptible individuals when they mount a harmful, adaptive immune response towards dietary gluten proteins. The only current treatment is a life‐long gluten‐free diet. All CeD patients have in their intestine proinflammatory CD4+ T cells that recognize posttranslationally modified gluten peptides in context of the disease associated MHC class II molecules HLA‐DQ2.5, DQ8, or DQ2.2.^[^ ^1^, ^2^ ^]^ Even on a gluten‐free diet, low numbers of gluten specific CD4+ T‐cells remain in the memory T‐cell compartment.^[^ ^3^ ^]^ These cells are rapidly reactivated when CeD patients eat gluten again.^[^ ^3^, ^4^ ^]^ CeD is therefore a life‐long condition that requires strict dietary measures. The proinflammatory T‐cell response to gluten results in small intestinal inflammation and mucosal destruction that causes clinical features such as diarrhea and nutrient deficiency. Mucosal affection is assessed from histological evaluation of small intestinal biopsies, where the morphology often is graded according to the categorical Marsh score.^[^ ^5^ ^]^ Marsh 0 represents normal morphology. Marsh 1 reflects normal morphology but with increased number of intraepithelial lymphocytes (IELs). Marsh 2 is similar to Marsh 1 but with addition of crypt hyperplasia. Marsh 3 has in addition blunting of the villi. Upon treatment with a gluten‐free diet, most patients recover with normalization of the intestinal mucosa to Marsh 0 or 1. Reintroduction of dietary gluten to treated patients can be done in the clinical contexts to confirm the diagnosis of CeD, and is used in clinical trials to evaluate drug efficacy where changes in intestinal morphology serve as read‐out. However, well‐treated CeD patients can respond very different to the same gluten challenge regime. This points to differences between patients that are not captured from routine clinical evaluation. Quantitative proteomic analysis of biopsy tissues allows for in‐depth and unbiased characterization, and can reveal differences that may be not be visible from histological evaluation. Here we have performed spatially resolved proteome analysis of intestinal biopsies collected before and after short‐term gluten challenge of 19 well‐treated CeD patients.^[^ ^6^ ^]^ Only some of the patients developed a strong mucosal response to gluten on day 14. We demonstrate that these patients have in their gut signs of ongoing, low‐grade antigluten immunity already before gluten challenge. These patients may therefore have a “head start” upon gluten challenge initiation. Our findings raise the question whether a standard gluten‐free diet is sufficient to fully curb the gluten‐specific immune response in all CeD patients.

We analyzed global protein expression in total tissue and laser capture microdissected epithelium from formalin fixed paraffin embedded biopsies collected before and after gluten challenge (**Figure** **1**A).^[^ ^6^ ^]^ All study participants were adults (>20 years old) with biopsy‐confirmed CeD that had followed a gluten‐free diet for more than 24 months (Table S1, Experimental Section, Supporting Information).^[^ ^6^ ^]^ Patients that participated in the trial were considered to be in complete remission with Marsh scores 0 or 1 and negative serum anti‐TG2 IgA.^[^ ^6^ ^]^ Nineteen of 20 enrolled patients completed the challenge, consuming one müsli bar with 5.7 g gluten protein per day for 14 days. Small intestinal biopsies were collected before challenge and on day 14. Biopsies collected from one patient before challenge was in a blinded reevaluation reclassified to Marsh 3.^[^ ^6^ ^]^ We collected tissue from the biopsy blocks for protein extraction and digestion followed by liquid chromatography and mass spectrometry for protein identification and label‐free quantification.^[^ ^7^ ^]^ From total tissue protein digests we quantified 4301 proteins (Figure S1, Supporting Information). Unsupervised clustering segregated samples from 8 biopsies of which seven were collected after challenge (Figure 1B). For the remaining patients, no clear separation was seen for biopsies collected before and after challenge (Figure 1B). Based on tissue proteome expression, we classified the patients as “responders” (*n* = 7) or “non‐responders” (*n* = 12) to the gluten challenge (Figure 1B; Table S1, Supporting Information). All five patients that developed Marsh 3 after challenge were “responders.” Gluten induced differential expression of 258 proteins (115 up, 143 down) in “responders” compared to 38 proteins in “non‐responders” (23 up, 15 down) (Figure 1C; Table S2, Supporting Information). Neutrophil‐derived and inflammatory proteins increased significantly in “responders,” while the majority of proteins with reduced expression were of presumed enterocyte origin. We therefore addressed how gluten challenge affected the intestinal epithelial compartment specifically. Epithelial cell layer samples spanning the entire crypt‐villus‐axis (≈2000 cells/sample) were isolated from tissue sections by laser capture microdissection followed by mass spectrometry‐based proteome analysis (Figures S2 and S3, Supporting Information). Based on the expression of 2404 proteins, epithelial samples separated as “responders” and “non‐responders” similar to total tissue samples (Figure 1D; Figure S3, Supporting Information). Baseline samples from “responders” also displayed skewing along principle component 1 (PC1) (Figure 1D). Gluten induced differential expression of 441 proteins in the “responder” epithelium (233 down, 208 up) compared to only 8 proteins in the epithelium of “non‐responders” (3 up, 5 down) (Figure 1E; Table S2, Supporting Information).

**Figure 1:**
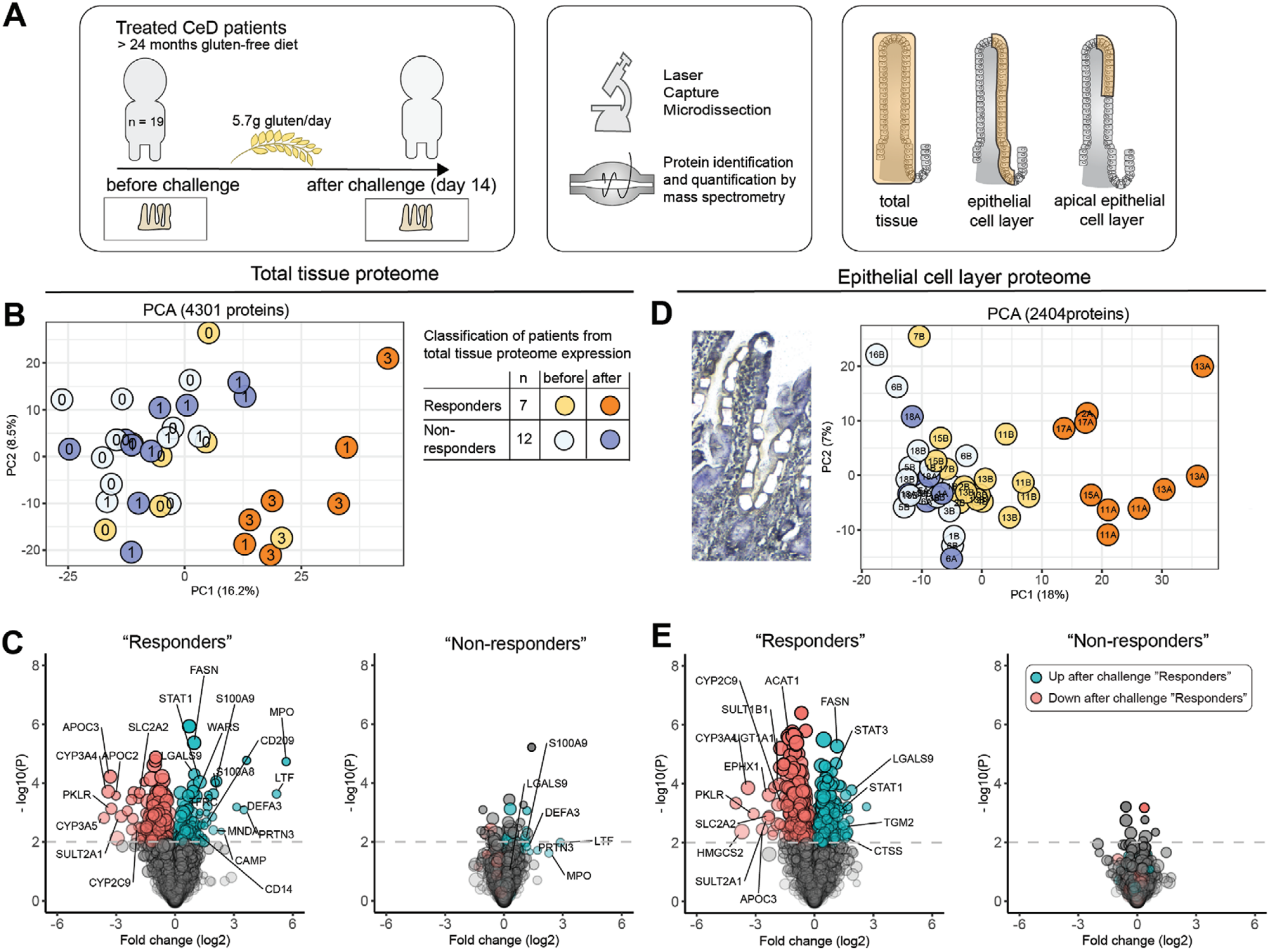
Tissue proteome analysis identifies CeD patients with strong mucosal response to gluten. A) Schematic presentation of the study. B) Principle component analysis (PCA) plot of total tissue samples based on expression of 4301 proteins. Each point represents one biopsy (*n* = 35). Number shows Marsh score for biopsy. C) Differential expression of total tissue proteins after versus before challenge. D) Principle component analysis plot of epithelial cell layer samples based on expression of 2404 proteins from two independent experiments. Each point represents one laser capture microdissected sample (*n* = 49 from 23 biopsies). Number indicates patient biopsy. Color indicates responder classification from (B). E) Differential expression of epithelial proteins after versus before challenge. C,E) Two‐sample Student *t*‐test, FDR = 0.05, Benjamini Hochberg adjusted. Significant proteins (*P* < 0.01) for “Responders” are indicated in blue (up) and red (down).

Unexpectedly, we observed larger difference between “responder” and “non‐responder” samples at baseline with skewed expression of many of the same proteins that changed in response to gluten (**Figure** **2**A,B; Table S2, Supporting Information). Biological pathways enriched in “responders” versus “non‐responders” at baseline correlated with biological pathways enriched in “responders” after versus before challenge (Figure 2C; Table S3, Supporting Information). “Shared Down” pathways reflect loss of intestinal absorptive function which agrees with reduced expression of several enterocyte proteins (Figure 2D; Table S3 and Figure S4, Supporting Information). “Shared Up” pathways represent innate immune activation, interferon/cytokine responses and complement/acute phase processes (Figure 2E; Table S3 and Figure S4, Supporting Information). Proteome analysis of laser capture microdissected samples from only the apical part of the epithelium from a subset of baseline biopsies separated "responders" and "non‐responders" (Figure 2F; Figure S5, Supporting Information) and confirmed an increase in complement cascade proteins in the epithelium of “responders” at baseline (Figure 2G; Figures S6 and S7, Supporting Information). These findings demonstrate that inflammatory processes, of which many are similar to those induced by gluten, are already ongoing in the intestinal epithelium of “responders” at baseline.

**Figure 2:**
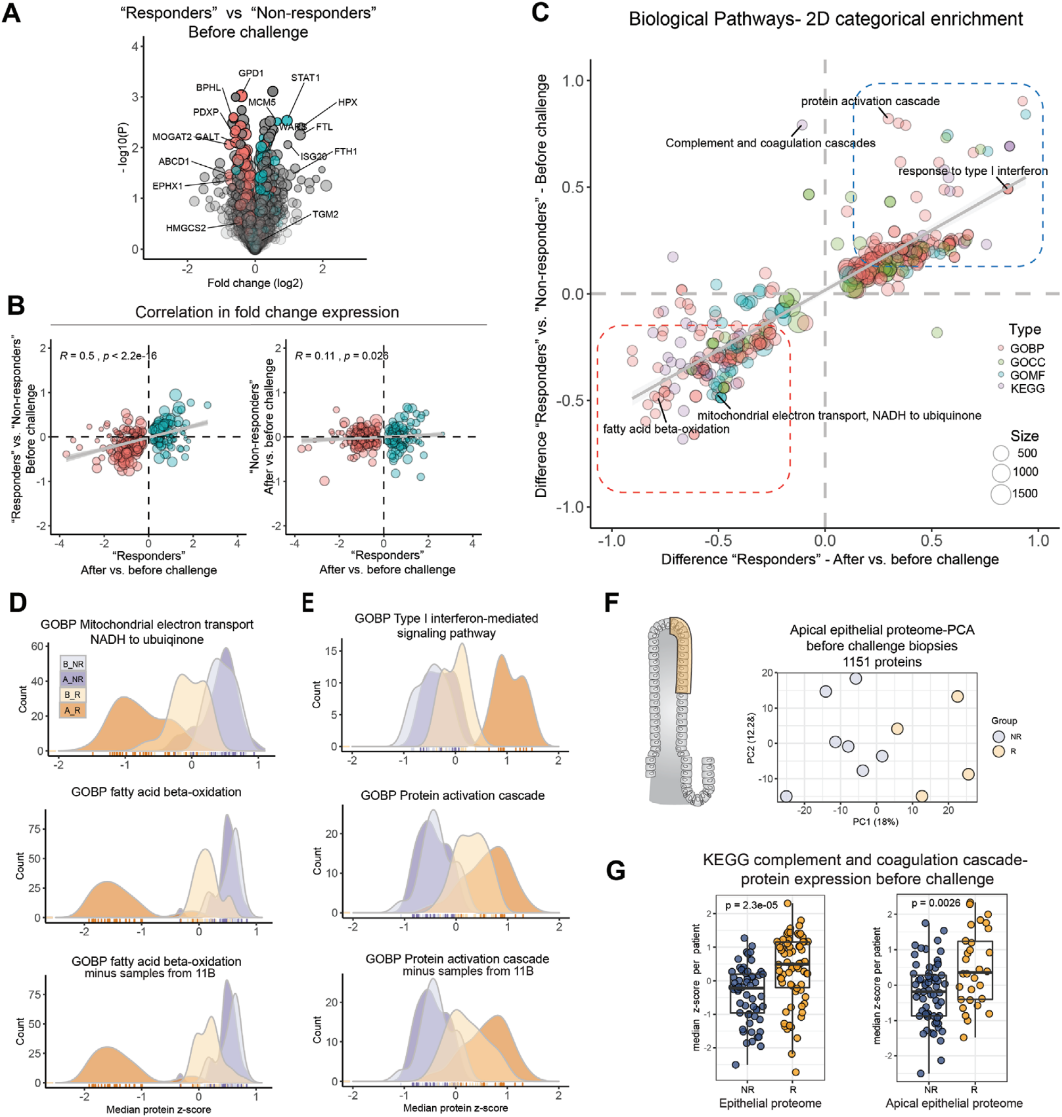
Patients with strong response to gluten have low‐level inflammation in the gut before challenge. A) Differential expression of epithelial proteins comparing “responder” and “non‐responder” biopsies at baseline. Color indicate significant proteins from Figure 1E (“Responders” after versus before challenge,*P* < 0.01, two‐sample Student *t*‐test, FDR = 0.05, Benjamini Hochberg adjusted). B) Pearson correlation of fold change protein expression for significant proteins (“Responders” after versus before challenge, *P* < 0.01). C) 2D‐categorical enrichment for biological pathways from *t*‐test fold change in expression. D,E) Comparison of expression for proteins that map to the indicated pathways (median *z*‐score per responder group). F) Principle component analysis (PCA) plot of apical epithelial cell layer samples based on expression of 1151 proteins. Each point represents one biopsy (*n* = 11). G) Expression of proteins that map to KEGG complement and coagulation cascade in baseline samples from the epithelial proteome and the apical epithelial proteome dataset (epithelial dataset; ten pathway proteins, each point represents median *z*‐scored protein expression per biopsy (*n* = 13); apical epithelial dataset, five pathway proteins, each dot represent *z*‐scored expression per sample (*n* = 19) from 11 biopsies)(Welch *t*‐test). A_R, after “responder”; B_R, before “responder”; A_NR, after “non‐responder”; B_NR, before “non‐responder”; R, “responder”; NR, “non‐responder”; GOBP, Gene Ontology Biological Processes; GOCC, Gene Ontology Cellular Compartment; GOMF, Gene Ontology Molecular Function; KEGG, Kyoto Encyclopedia of Genes and Genomes

The small intestinal lesion in CeD is characterized by epithelial changes such as increased frequency of IELs, crypt hyperplasia, and villous blunting. Villous height to crypt depth (Vh:Cd) ratio is a continuous morphometric variable that, similar to the Marsh score, can reflect gluten‐induced pathology.^[^ ^8^ ^]^ The majority of cells in the epithelium are absorptive enterocytes that come from stem cells in the proliferative crypts that divide and mature as they migrate up to the villi tips. Inflammation, increased epithelial turnover, and crypt hyperplasia as occurs in CeD results in fewer mature enterocytes which affects intestinal function. We mapped our proteome data to single cell RNA‐sequencing‐derived gene‐sets for “mature enterocytes” and “goblet cells” to compare cell‐type protein expression with histological measures (Table S4, Supporting Information).^[^ ^9^, ^10^ ^]^ “Mature enterocyte” proteins showed lowest expression in biopsies from “responders” after challenge, with an opposite expression pattern for “goblet cell” proteins (**Figure** **3**A,B; Figure S8, Supporting Information). Median expression of “mature enterocyte” proteins showed a positive correlation with Vh:Cd ratio previously measured on the same biopsies while “goblet cell” protein expression showed negative correlation Vh:Cd ratio (Figure 3C,D). These plots show clearly that “responders” differ from “non‐responders” already at baseline. These baseline differences were captured by the Vh:Cd ratio (Figure 3E), but not by the categorical Marsh score (Figure 3F) or IEL frequency (Figure 3G). Thus, the patients that respond strongly to gluten have altered epithelial protein expression at baseline, with activation of crypt cells in the absence of IEL infiltration.

**Figure 3:**
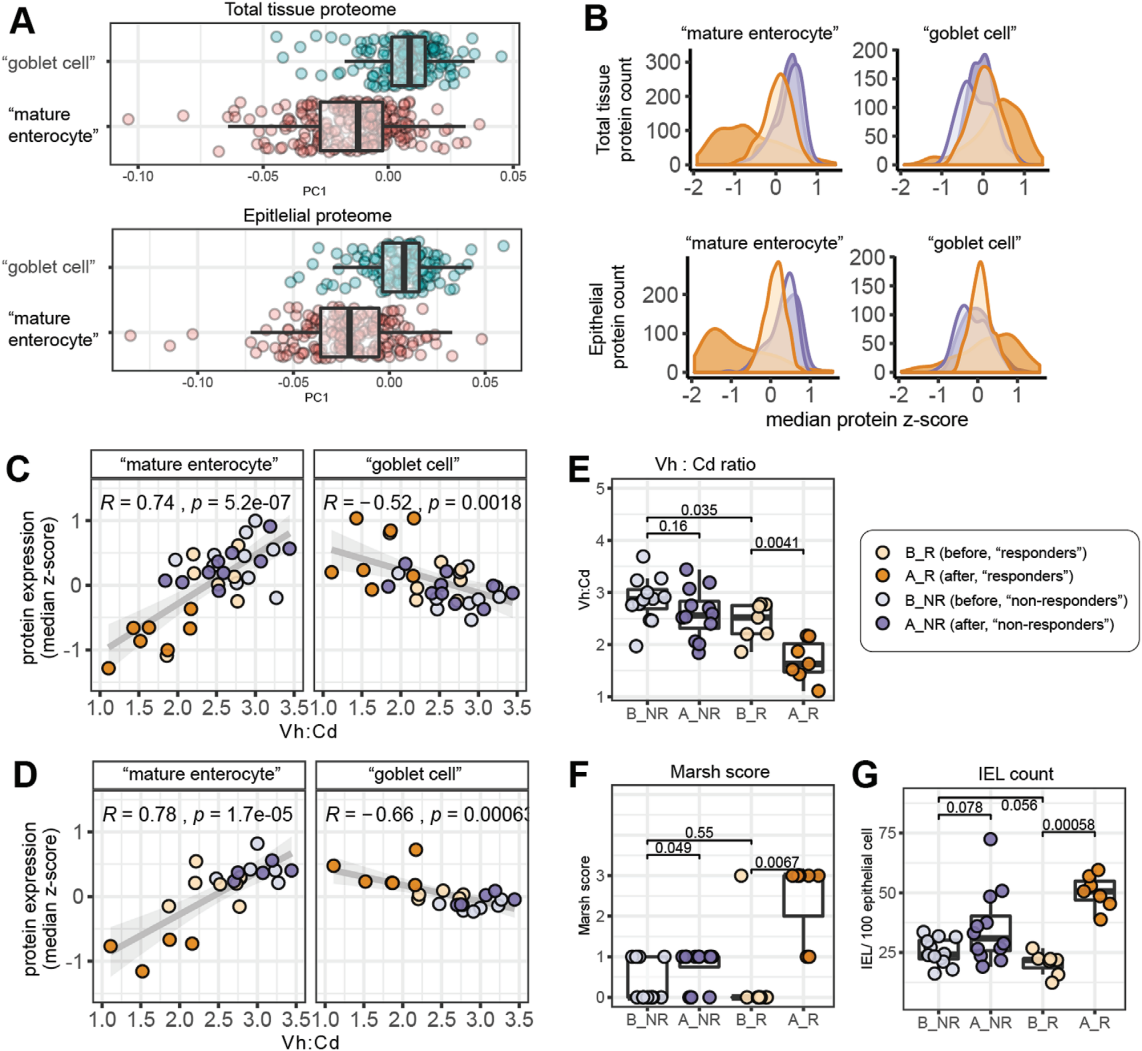
Epithelial cell‐type protein expression indicate mild crypt hyperplasia in “responders” at baseline. A) Position of proteins mapped to “mature enterocytes” and “goblet cells” along principle component 1 (PC1) in total tissue and epithelial proteome dataset. B) Expression of cell‐type specific proteins (median *z*‐score per responder group). C,D) Correlation (Pearson) between "mature enterocyte" and "goblet cell" protein expression (median *z*‐score per patient) with Vh:Cd ratio measured on the same biopsies from total tissue (C) and epithelial proteome (D) datasets (E–G). Comparison of E) Vh:Cd ratio F) Marsh score, and G) IEL count between responder groups(Pairwise comparison by Mann‐Whitney U test).

We next asked whether available blood parameters measured at baseline before the gluten challenge reflected this low‐level of mucosal inflammation. Serum ALAT, ALP, ASAT, CRP, ferritin, GT, Hb, and plasma TNF‐*α* were all slightly higher in “responders” compared to “non‐responders” while serum transferrin was lower in “responders” (**Figure** **4**A; Table S1 and Figure S9, Supporting Information). Although modest, this clinical biochemistry profile supports the notion of low‐level inflammation in “responders.” The mucosal damage in CeD is considered to be a direct consequence of the adaptive immune response to gluten. Gluten specific CD4+ T cells as detected with HLA‐DQ:gluten peptide tetramers have previously been quantified in blood, and in intestinal biopsies from a subset of patients from this gluten challenge study (Table S1, Supporting Information).^[^ ^3^, ^6^ ^]^ “Responders” had higher frequency of gluten specific CD4+ T cells in the gut and in the blood at baseline compared to “non‐responders” (Figure 4B). This difference was not explained by the duration of gluten‐free diet prior to gluten challenge (Figure 4C). Frequency of gluten specific CD4+ T cells in the gut at baseline correlated with “mature enterocyte” protein expression, with separation of “responders” and “non‐responders” (Figure 4D, top left panel). Weaker correlation was observed between “mature enterocyte” protein expression and blood T‐cell frequency at baseline, and between Vh:Cd ratio and gut or blood T‐cell frequencies (Figure 4D). These data indicate that “responder” patients with ongoing low‐level inflammation at baseline also have more gluten‐specific CD4+ T cells in particular in the gut at baseline, despite long‐term treatment with gluten‐free diet.

**Figure 4:**
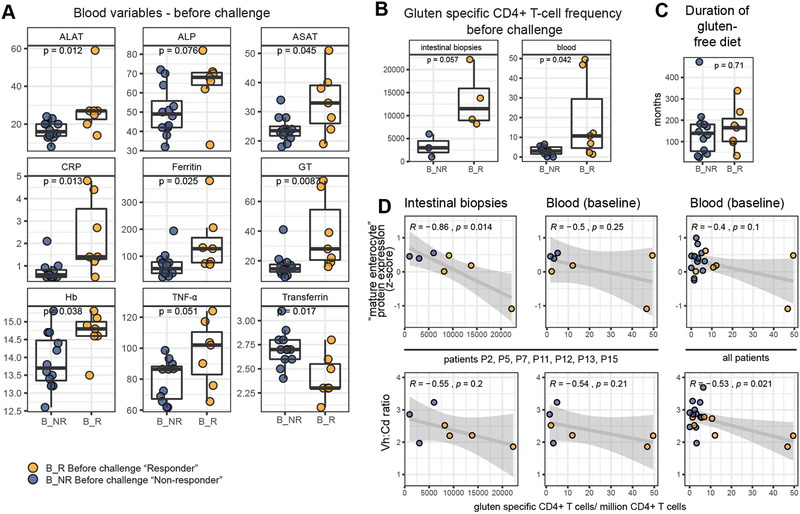
Presence of low‐level serum inflammation and antigluten immunity at baseline in "responder" patients. A) Comparison of clinical biochemistry and cytokines measured at baseline before challenge for “responders” and “non‐responders” (Mann‐Whitney U test). B) Frequency of gluten specific CD4+ T‐cells per million CD4+ T‐cells from gut biopsies collected at baseline (left, P2, P5, P7, P11, P12, P13, and P14)^[^ ^3^ ^]^ and gluten‐specific gut homing effector memory T‐cells per million CD4+ T‐cells in blood at baseline.^[^ ^6^ ^]^ (Mann‐Whitney U test). C) Duration of gluten‐free diet before gluten challenge. D) Correlation between mature enterocyte protein expression (from total tissue data) (top row) or Vh:Cd ratio (bottom row) with gluten‐specific CD4+ T‐cell frequencies at baseline in gut (left) and blood (middle) for patients P2, P5, P7, P11, P12, P13, and P14, and blood (right) for all patients (Pearson correlation).

In this study we show that quantitative proteome analysis of routine histology biopsies reveals differential presence of low‐level inflammation in the intestine of CeD patients considered to be well‐treated by gluten‐free diet. An RNASeq study of treated CeD patients undergoing gluten challenge also found differences between patients before and after gluten challenge.^[^ ^11^ ^]^ The patients in our study that developed clear mucosal response by day 14 of the gluten challenge had ongoing low‐level inflammation at already at baseline. Gluten specific CD4+ T cells peak in blood on day 6 after gluten intake.^[^ ^12^ ^]^ From the current gluten challenge, 12 of the 15 patients that were analyzed on day 6 of challenge showed more than twofold increase in gluten specific CD4+ T‐cells.^[^ ^6^ ^]^ However this relative increase hides differences in baseline cell numbers, which is likely to be a more crucial variable for development of mucosal pathology. The T‐cell autocrine cytokine IL‐2 is secreted by gluten specific CD4+ T cells upon intake of gluten and is now considered the major driver of immediate gastrointestinal symptoms to gluten in CeD patients.^[^ ^13^ ^]^ Also in patients from the current study, mean fold increase in plasma IL‐2 within <6 h after gluten intake correlated with baseline frequency of gluten‐specific CD4+ T cells in blood.^[^ ^13^ ^]^ Thus, development of symptoms and mucosal pathology in response to gluten is linked to the magnitude of antigluten immunity present at the time of gluten consumption. Conceivably, mucosal pathology may develop more uniformly in response to longer gluten challenge regimes (e.g., ten weeks), where the gluten specific CD4+ T‐cells have time to expand sufficiently in all patients.

Proteins with a clearly altered expression after a 14‐day gluten challenge are already minutely altered in the “responders" at baseline. The protein changes are associated with the presence of gluten‐specific CD4+ T cells in the gut mucosa. This suggests ongoing gluten immunity in these patients even in absence of mucosal changes as detectable by routine histology. Our findings suggest that in some well‐treated celiac patients on a standard gluten‐free diet there is ongoing antigluten immunity.

## Experimental Section

The gluten challenge trial has previously been described by Sarna et al.^[^ ^6^ ^]^ Detailed Experimental section for the current study is found in the Supporting Information. MS data have been deposited to the ProteomeXchange Consortium^[^ ^14^ ^]^ via the PRIDE partner repository with the dataset identifier PXD018977.

##### Ethical Approval

The study was approved by the regional ethical committee of South‐east Norway (ref 2013/1237) and patients had given written consent to perform the gluten challenge study.^[^ ^6^ ^]^ The trial was registered at ClinicalTrial.gov, NCT02464150.

## Conflict of Interest

KEAL has privately or via his employer been a consultant during the two last years for Chugai Pharmaceutical, Bioniz Therapeutics, Interexon Actobiotics, Amyra Biotech AG and Dr. Falk Pharma GMBH. LMS has privately or via his employer been a consultant during the two last years for Amagma Therapeutics, Chugai Pharmaceutical, Bioniz Therapeutics, Interexon Actobiotics, UCB Biopharma, Merck and GSK. The other authors declare no conflicts of interest.

## Supporting information

Supporting Information

Supplemental Table 2

Supplemental Table 3

Supplemental Table 4
