# Supplementary material for: In Well‐Treated Celiac Patients Low‐Level Mucosal Inflammation Predicts Response to 14‐day Gluten Challenge
Source: Adv Sci (Weinh). 2021 Jan 4;8(4):2003526. doi: 10.1002/advs.202003526 (PMC7887593; doi:10.1002/advs.202003526)
Supplement: Supplementary file 1 — Supporting Information [file ADVS-8-2003526-s001.pdf]

© 2020 Wiley-VCH GmbH

ADVANCED SCIENCE
Open Access

Supporting Information

for *Adv. Sci.,* DOI: 10.1002/advs.202003526

In well-treated celiac patients low-level mucosal inflammation predicts response to 14-day gluten challenge

Jorunn Stamnaes, Daniel Stray, Maria Stensland, Vikas K. Sarna, Tuula A. Nyman, Knut E. A. Lundin, Ludvig M. Sollid **

In well-treated celiac patients low-level mucosal inflammation predicts response to 14-day gluten challenge

Jorunn Stamnaes, Daniel Stray, Maria Stensland, Vikas K. Sarna, Tuula A. Nyman, Knut E. A. Lundin, Ludvig M. Sollid**

**Corresponding authors: jorunn.stamnas @medisin.uio.no, l.m.sollid@medisin.uio.no*

Supporting Information

Supplementary Materials and methods

Figure S1 Total tissue quality control

Figure S2 Laser capture microdissection (LCM) method validation

Figure S3 LCM epithelial samples batches and quality control

Figure S4 Differential expression at baseline for proteins that map to enriched biological pathways

Figure S5 LCM apical epithelial samples quality control

Figure S6 Expression of individual proteins in complement cascade

Figure S7 Expression of hemoglobin

Figure S8 Correlation between cell-type protein expression in total tissue and laser capture isolated epithelial proteome data

Figure S9 Reference limits of blood variables

Table S1 Patient info and data

Table S2 T-test proteins total tissue and LCM isolate epithelium (separate xslx.file)

Table S3 Enriched pathways- 2D enrichment (separate xlsx.file)

Table S4 List of mature enterocyte and goblet cell specific proteins (separate xlsx.file)

Supplementary Materials and Methods

Patient cohort and clinical information

The patient cohort subjected to 14-day oral gluten challenge has previously been described by Sarna et al. where full clinical information can be found.[1] For this study we have used material from formalin fixed paraffin embedded (FFPE) small intestinal biopsies from 19 of 20 enrolled patients that completed the challenge. Biopsies were collected at baseline before challenge and at day 14 of challenge. Histomorphometry (Marsh score and villous height to crypt depth (Vh:Cd) ratio) and intraepithelial lymphocyte (IEL) counts was previously reported and were performed on sections from the same FFPE biopsy blocks as used in this study. All patients were upon study initiation considered to be in complete clinical and mucosal remission at baseline before challenge as assessed by Marsh score and serum anti-transglutaminase IgA titers. One patient (P11, CD442) was initially evaluated to be in mucosal remission before onset of gluten challenge, but was later revised to "Marsh 3" after a blinded re-evaluation of all biopsies. Clinical biochemistry and cytokine measurements were performed on baseline blood samples. Frequencies of CD4+ gluten specific T cells in blood (baseline for all patients and at day 6 for 15 patients) and gut (baseline and day 14 for 7 patients) have previously been reported.[1, 2]

Total tissue sample digestion and processing

To generate total tissue digests, fifteen 5 μm thick sections from each FFPE biopsy block were collected in a tube (Eppendorf, Hamburg, Germany) and dehydrated by 10 min incubations in 80 %, 96 % and 100 % ethanol, respectively, followed by dewaxing by two incubations with xylene (2x3 min at 55 °C) and two incubations in 100 % ethanol (3 min at room temperature). Dewaxed

tissue was resuspended in 20 µL 50 mM ammonium bicarbonate with 0.2 % ProteaseMAX surfactant (Trypsin enhancer; Promega, Madison, WI) followed by addition of dithiotreitol (1µL 0.5 M) and ammonium bicarbonate (73.5 µL 50 mM). Formalin crosslinks were cleaved by heating the samples (98°C 90 min) followed by sonication (60 min in a water bath). Protein amount was estimated by DirectDetect (Millipore, Merck, Darmstadt, Germany). Disulfide bonds were reduced with dithiotreitol (1 µL 0.5 M per sample, 20 min incubation with gentle agitation at 56°C) and alkylated with iodoacetamide (2.7 µL 0.55 M per sample, 15 min incubation with gentle agitation at room temperature in the dark). To digest proteins, ProteaseMAX (2 µL 1%) and Trypsin (1 µg) (Sequencing grade, ProMega) was added to each sample followed by incubation in a wet chamber over night at 37°C. Peptides were purified on C18 micro columns as previously described.[3] Purified samples were adjusted to a final volume of 11 µL or 20 µL depending on protein concentration.

LCM sample collection and processing

Eight µm tissue sections were adhered to PEN-covered slides (Zeiss) and dried at 37°C. Dry sections were dewaxed in xylene (3 min + 2 min) followed by 1 min in 100 % ethanol, 95 % ethanol and 70 % ethanol, respectively followed by 2 x 1 min in water. Tissue was visualized by staining with Mayer's hematoxylin solution (Sigma) for ~30 s followed by rinsing in tap water. Stained sections were air-dried and stored dry until cutting. Samples were collected using a PALM MicroBeam laser capture microdissection system (Carl Zeiss MicroImaging, Munich, Germany), and isolated tissue collected in 0.5 mL opaque adhesive cap tubes (Zeiss). To validate our LCM approach and confirm that we could obtain region specific protein expression data we collected samples from three distinct tissue regions for comparative analysis: Two samples were

collected per region from lamina propria, villus and crypt epithelium. For total epithelial cell layer analysis on average 250 000 μm^2 tissue was collected per sample, while for apical epithelial tissue from baseline biopsies, on average 150 000 μm^2 tissue was collected per sample.

Two cohorts of total epithelial cell layer samples spanning the entire crypt villus axis were collected (LCM1; 17 samples from 13 biopsies; LCM2: 25 samples from 20 biopsies). For apical epithelial cell layer analysis, 24 samples from 12 biopsies before challenge were collected. Dissected tissue was retrieved from adhesive caps using ammonium bicarbonate (10 μL 50 mM) with ProteaseMax Surfactant (0.2 %) followed by ammonium bicarbonate (10 μL 50 mM) and transferred to 0.5 mL Low-Bind tubes (Eppendorf, Hamburg, Germany). Samples were heated to 98°C for 90 min followed by sonication in water bath for 60 min. Disulfide bridges were reduced by addition of dithiotreitol (2 μL 0.1 M) followed by incubation for 20 min at 56°C and alkylated by addition of iodoacetamide (2 μl 55 mM) followed by incubation for 15 min in the dark at room temperature. Samples were digested by addition of trypsin (1.5 μL 0.01 g L^{-1}) and incubation in wet chamber over night at 37°C. Peptides were purified on C18 micro columns and eluted samples were adjusted to a final of 7 μL with 0.1 % formic acid.

Mass spectrometry analysis

Three μL digest was injected per run for all samples. Total tissue digests were analyzed with two technical replicates. Digested LCM1 samples were analyzed together (LCM cohort 1, 17 samples from 13 biopsies). Digested LCM2 samples were run together with 13 samples from LCM1 resulting in total 38 samples from 21 biopsies in the LCM2 cohort 2 dataset (LCM cohort 2). Compartment samples (lamina propria, epithelial villi and crypts) were run together with LCM

cohort 2 samples. All experiments were performed on an Easy nLC1000 nano-LC system connected to a quadrupole - Orbitrap (QExactive Plus) mass spectrometer (ThermoElectron, Bremen, Germany) equipped with a nanoelectrospray ion source (EasySpray/Thermo). For liquid chromatography separation we used an EasySpray column (C18, 2 µm beads, 100 Å, 75 µm inner diameter) (Thermo) capillary of 50 cm bed length. The flow rate used was 0.3 µL min^{-1}. For total tissue samples, the solvent gradient was 2% to 5 % in 10 min, to 19% in 170 min and then to 35% B in 60 min followed by a wash with 90% B for 20min. For LCM isolated sample, the solvent gradient was 2 - 7% B in 10 min, then to 30 % B in 55 min and finally a wash with 90 % B in 20 min. Solvent A was aqueous 0.1 % formic acid, whereas solvent B was 100 % acetonitrile in 0.1 % formic acid. Column temperature was kept at 60ºC.

The mass spectrometer was operated in the data-dependent mode to automatically switch between MS and MS/MS acquisition. Survey full scan MS spectra (total tissue: m/z 300 to 1,500; LCM samples: *m/z* 400 to 1,200) were acquired in the Orbitrap with resolution R = 70,000 at *m/z* 200 (after accumulation to a target of 3,000,000 ions in the quadruple). The method used allowed sequential isolation of the most intense multiply-charged ions, up to ten, depending on signal intensity, for fragmentation on the HCD cell using high-energy collision dissociation at a target value of 100,000 charges or maximum acquisition time of 100 ms. MS/MS scans were collected at 17,500 resolution at the Orbitrap cell. Target ions already selected for MS/MS were dynamically excluded for 30 seconds. General mass spectrometry conditions were: electrospray voltage, 2.1 kV; no sheath and auxiliary gas flow, heated capillary temperature of 250°C, normalized HCD collision energy 25%.

Mass spectrometry data have been deposited to the ProteomeXchange Consortium [4] via the PRIDE partner repository with the dataset identifier PXD018977

Protein identification, quantification and data analysis

MS raw files were processed in the MaxQuant environment [5] (version 1.6.1.0) with the integrated Andromeda search engine [6] for peptide and protein identification, with a FDR threshold of 0.01 for peptide and for protein identification. The human UniProtKB FASTA database (September 2018) was used as forward database for protein identification. Match between runs was enabled and label-free protein quantification (LFQ) was performed using the MaxQuant's Label Free Quantification algorithm with a minimum ratio count of one. [7] Methionine oxidation and N-terminal acetylation was used as variable modification and carbamidomethyl cysteine as fixed modification. We performed separate MaxQuant searches for MS data from total tissue, total epithelial LCM cohort 1, total epithelial LCM cohort 2 (including compartment samples) and apical epithelial LCM samples. MaxQuant results were processed in Perseus (version 1.6.2.2). [8] For all datasets, proteins matched to the reverse decoy database, identified by site or identified as potential contaminant were removed. Poor quality samples were removed based on number of LFQ valid values as shown in Figure S1, Figure S3 and Figure S5.

For the total tissue dataset, technical replicates were averaged and the dataset filtered to keep only proteins with valid LFQ-values in at least 70% of the samples in at least one of four sample-groups (responders or non-responders before or after challenge). Missing values were imputed

based on normal distribution to simulate low abundant LFQ values. Samples with <3600 LFQ valid values were removed from the dataset (samples from biopsies 1B, 6A, 14A).

Protein identification and label-free quantification was performed separately for LCM cohort 1 and LCM cohort 2 to avoid excessive reliance on data extrapolation from match-between-runs. Samples were filtered based on LFQ valid values (LCM cohort 1 <2000, one sample removed giving final 16 samples from 13 biopsies; LCM cohort 2 <2600, 5 samples removed resulting in data from 33 samples from 21 biopsies for analysis). LCM cohort 1 and LCM cohort 2 datasets were filtered separately to keep only proteins with valid LFQ-values in at least 70% of the samples in at least one of four sample-groups, followed by missing value imputation as described for total tissue samples. LFQ values from LCM cohort 1 and LCM cohort 2 datasets were merged by matching protein groups from LCM cohort 1 (n = 2495) to protein groups from LCM cohort 2 (n = 3080) (total 49 samples from 23 biopsies). Only protein groups present in both datasets (n = 2404) were kept, and PC1 was subtracted from the combined LFQ values prior to downstream analysis of the total epithelial proteome dataset.

For the apical epithelial LCM dataset, samples with less than 700 LFQ valid values were removed (5 samples). Data were filtered for protein groups present in 50 % of responder or non-responder samples followed by imputation of missing values, resulting in expression values for 1151 proteins across 19 samples from 11 biopsies.

2D categorical enrichment of biological pathways

Biological pathway enrichment analysis was performed in Perseus (2D enrichment based on student *t*-tests fold difference) [9]. Enrichment data for Gene Ontology pathways (GO Biological Process, GO Cellular Compartment, GO Molecular Function) and Kyoto Encyclopedia of Genes and Genomes (KEGG) pathways were exported and visualized in R. Enriched pathways were filtered for pathways with <100 genes and Student *t*-test fold difference (<-0.25 or >0.25) for both "responders" after vs. before challenge and "responders" vs. "non-responders" before challenge (Shared Down and Shared Up). Shared Up were 38 pathways of which 23 were GO Biological Processes (from 410 unique proteins). Shared Down consisted of 77 pathways of which 42 were GO Biological Processes (from 573 unique proteins). Expression of proteins that mapped to GO Biological Processes in Shared Up or Down were compared between responder groups (ANOVA and Tukey's honest significance test, FDR = 0.05) (**Table S3**). Z-scored protein expression (median expression per responder group) was visualized for selected pathways.

Cell-type gene set annotation

Small intestinal epithelial cell-type gene-sets were retrieved from [10]. Mouse genes were converted to human gene orthologs (www.ensembl.org/biomart) followed by manual curation of the lists. From the "mature enterocyte" protein list (n =497) 222 proteins were present in the total tissue dataset and 181 proteins were present in the epithelial dataset. From the "goblet cell" protein list (n = 401) we found 157 proteins in the total tissue dataset and 119 proteins in the epithelial dataset (**Table S4**).

Statistical analysis

Protein LFQ values were log_2 normalized before analysis. Details on quality control of mass spectrometry datasets, filtering of low quality samples, filtering of proteins and imputation of missing values is explained in the section "Protein identification, quantification and data analysis". All statistical analysis and data visualization was performed in Perseus [11] (version 1.6.2.2) or the R framework (R version 3.6.1, https://www.r-project.org/). To address differential protein expression, technical replicates (total tissue) or biological replicates (LCM samples) were averaged to give median protein expression per biopsy before comparison by two-sample Student t-test (FDR = 0.05, Benjamini Hochberg adjustment for multiple testing) (**Table S2**). Clinical and serological variables were compared by Mann-Whitney U test with no correction for multiple testing. Expression of individual proteins or proteins mapped to biological pathway were compared between patient groups by Welch t-test or ANOVA and Tukey's honest significance test (FDR = 0.05). Pearson correlation was used to compare cell-type protein expression (median z-scored expression per biopsy) with Vh:Cd ratio or gluten-specific CD4+ T cell frequencies.

Supplementary Figures

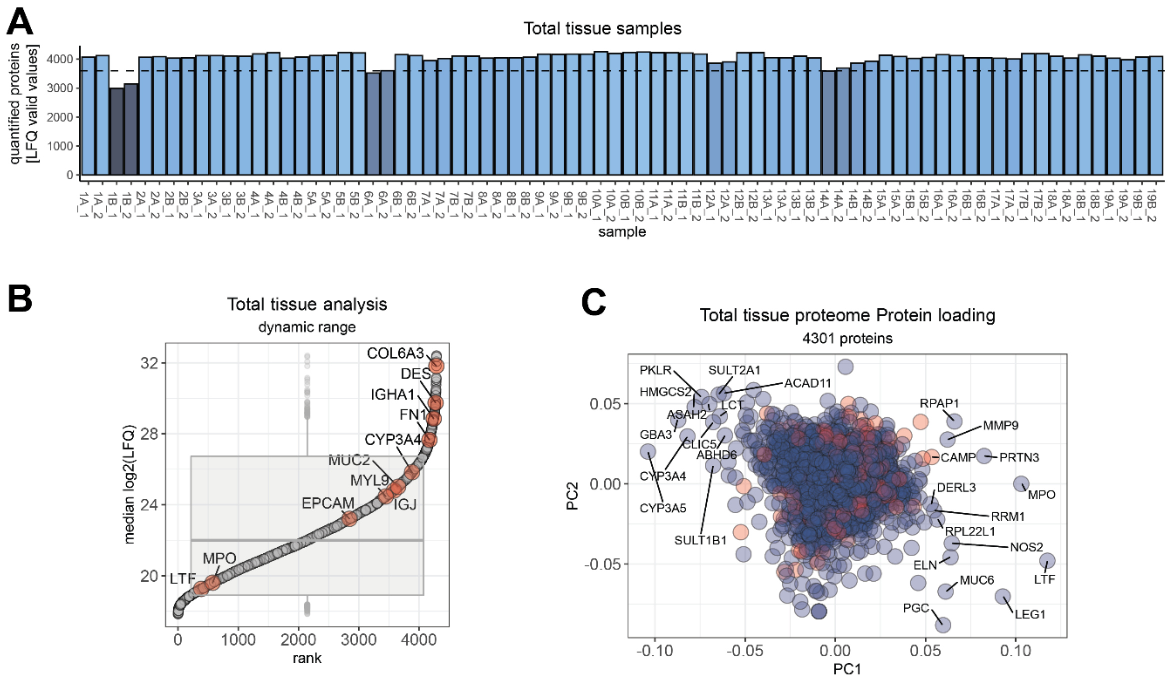

Figure S1. LC-MS/MS analysis of total tissue digest from biopsies collected before and after challenge

A. Samples analyzed by LC-MS/MS from total tissue digests. Samples with <3600 valid values from LFQ protein quantification were removed from the dataset before analysis (samples from biopsies 1B, 6A, 14A) **B**. Dynamic range of LFQ quantified protein groups from total tissue digest samples. Boxplot denotes 5% and 95% quantile and error bars 1% and 99% quantile. **C**. Protein loadings that drive separation of samples in Figure 1B. Color shows number of peptides used for protein quantification (blue = razor + unique >1, n = 3963; red; razor + unique = 1, n = 338)

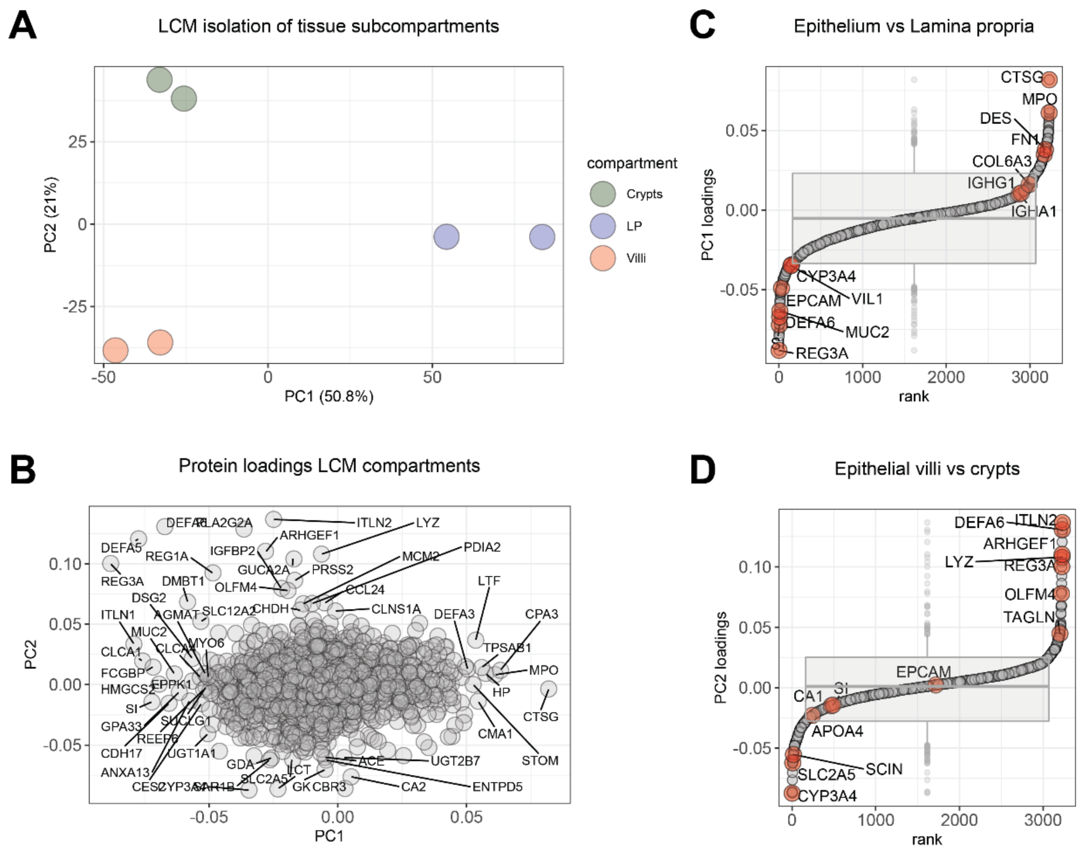

Figure S2. Validation of LCM approach by analysis of distinct tissue sub-compartments

A. PCA analysis plot of six samples collected from three distinct tissue compartments (Epithelial villi = red; Epithelial crypts = green; Lamina Propria = blue). **B**. Protein loadings that drive the separation in A. **C**. Protein distribution along PC1 that separates epithelium from lamina propria. Selected proteins representative for intestinal epithelium and lamina propria are indicated in red **D**. Protein distribution along PC2 that separates total epithelium from crypts. Selected proteins representative for enterocyte absorptive function (villi) and epithelial crypt proliferation and Paneth cells (crypts) are indicated in red.

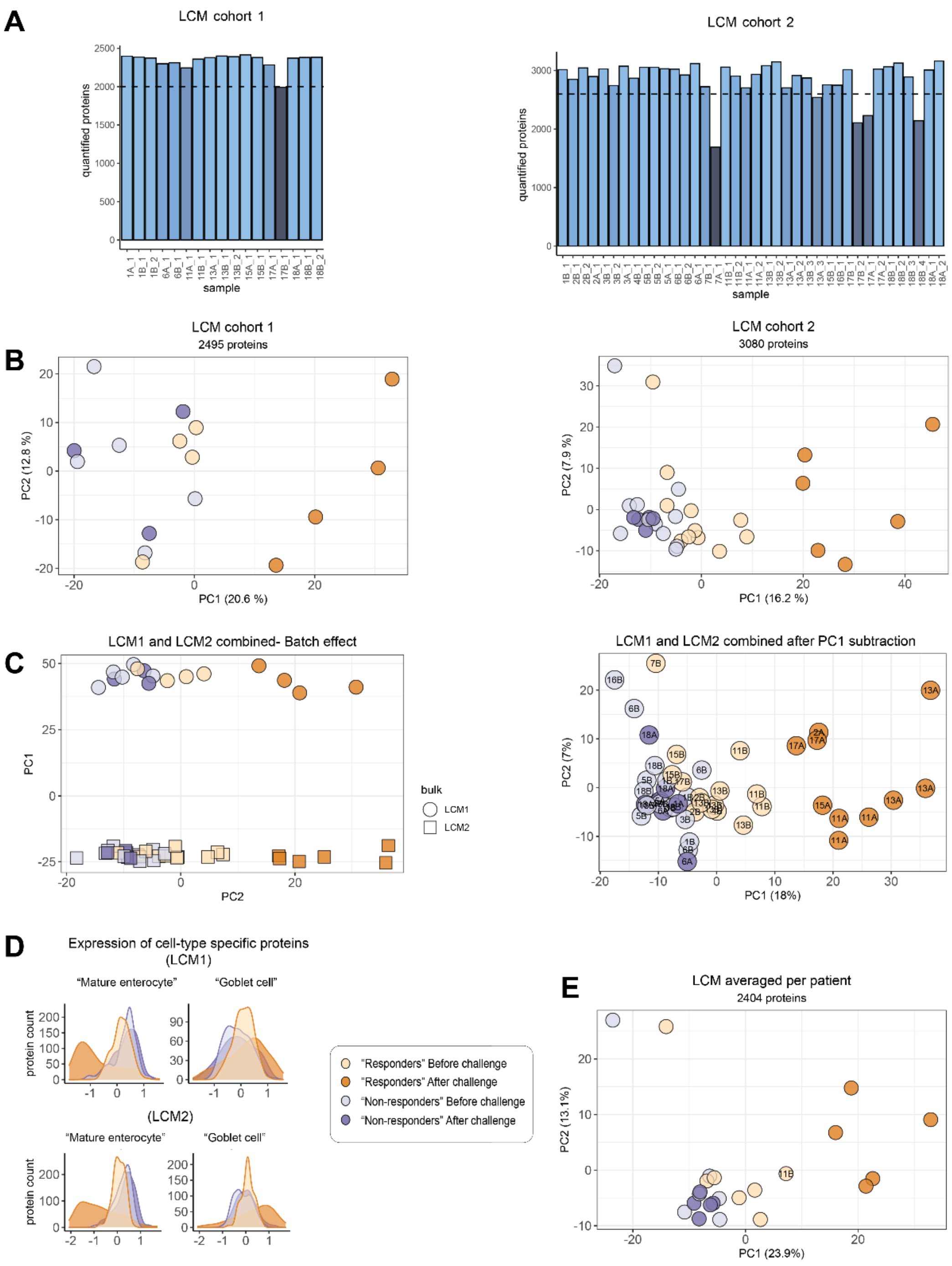

Figure S3. LCM isolation of epithelial cell layer samples from biopsies collected before and after gluten challenge

A. Number of proteins with LFQ values (quantified proteins) for each sample in the two analysis cohorts of LCM isolated epithelium. Protein identification and label-free quantification was

performed separately for the two cohorts to avoid excessive reliance on data extrapolation from match-between-runs. Samples were filtered based on LFQ valid values (LCM1 <2000, one sample removed before analysis; LCM2 <2600, 5 samples removed before analysis) **B**. PCA plot show distribution of samples from the independently analyzed LCM cohorts. **C** Data from LCM1 and LCM2 was combined (left) and PC1 subtracted. The resulting PCA plot based on expression of 2404 proteins (right) shows similar distribution of individual samples as observed for total tissue digest (n = 49 from 23 biopsies) . **D**. The distribution of cell-type specific proteins is similar for both LCM cohorts. **E**. PCA plot from median protein expression per biopsy (compared to distribution of all samples as shown in C)

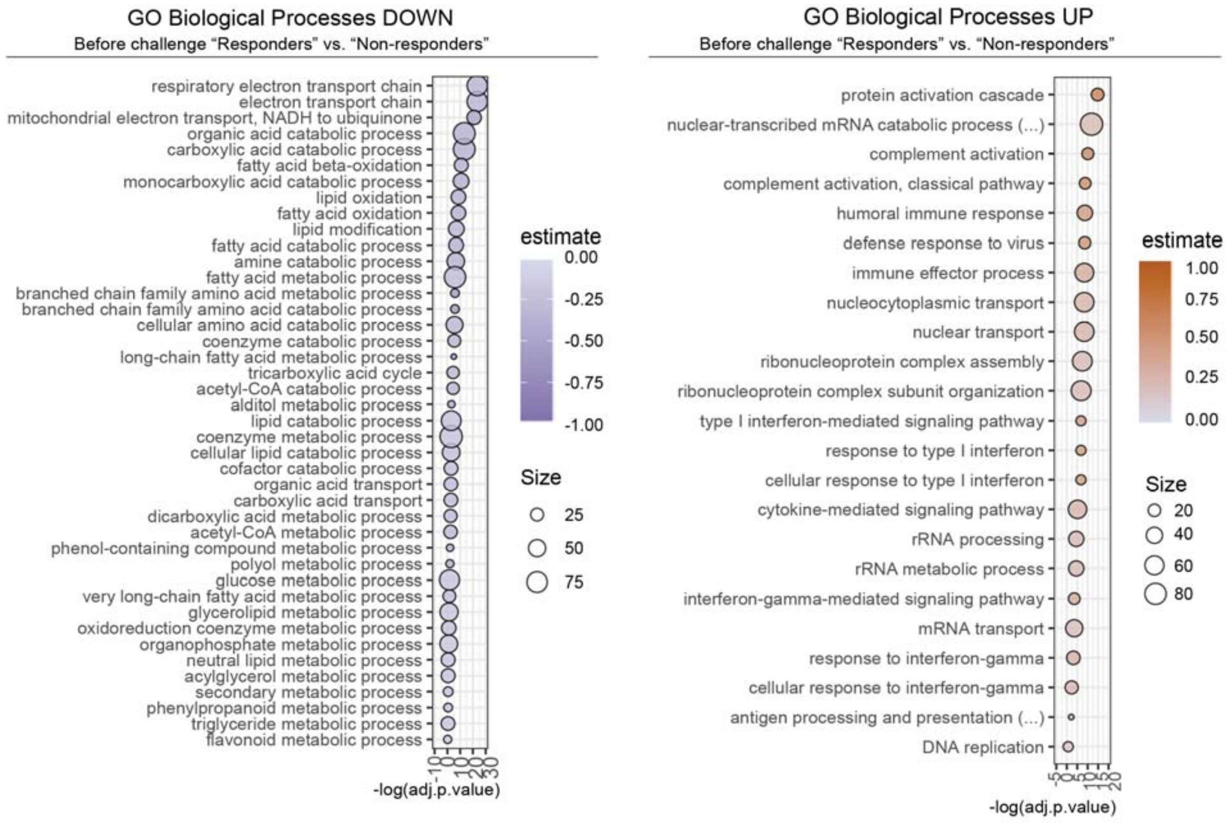

Figure S4 Differential protein expression at baseline among enriched biological pathways

Differential expression of pathway proteins was compared between all groups by ANOVA and Tukey's honest significance test (FDR = 0.05). Shared Down and Shared Up GO Biological Processes were ranked according to -log adjusted P-value comparing "responders" and "non-responders" (NR) before challenge. Color indicate difference in expression (estimate) and size show number of proteins mapped to the pathway.

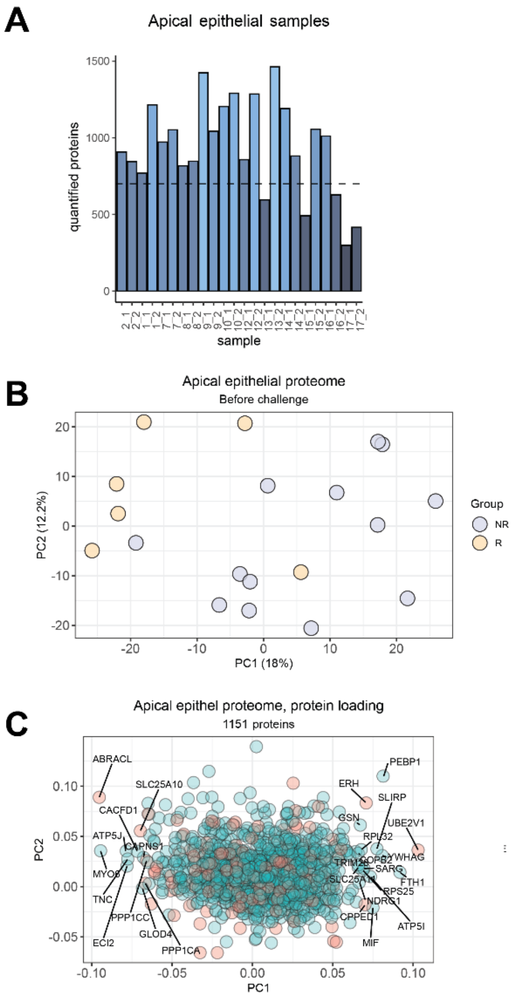

Figure S5 Quality control of apical epithelial proteome dataset A. Number of proteins with LFQ valid values (quantified proteins) for the apical epithelial samples (n = 24, from 12 biopsies). Samples with valid values <700 were removed from the dataset before analysis (5 samples). **B**. PCA plot show distribution of all samples after filtering (n = 19 from 11 biopsies) **C**. Protein loadings that drive separation in B (blue, razor + unique peptides >1, red = razor + unique = 1).

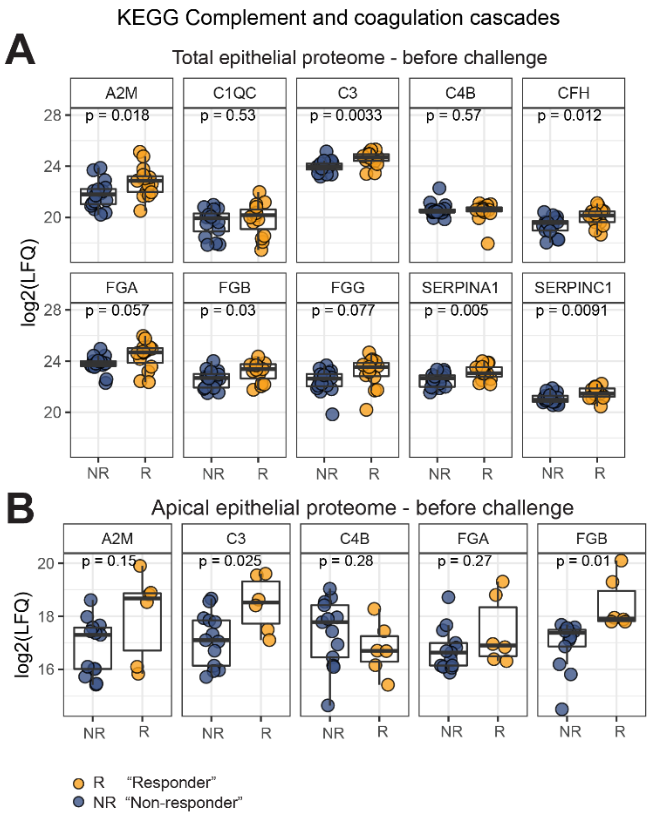

Figure S6 Expression of individual proteins that map to KEGG complement and coagulation cascades pathway Expression of individual proteins from microdissected samples from baseline biopsies. (**A**) Total epithelial proteome and (**B**) apical epithelial proteome. (Comparison by Welch t-test).

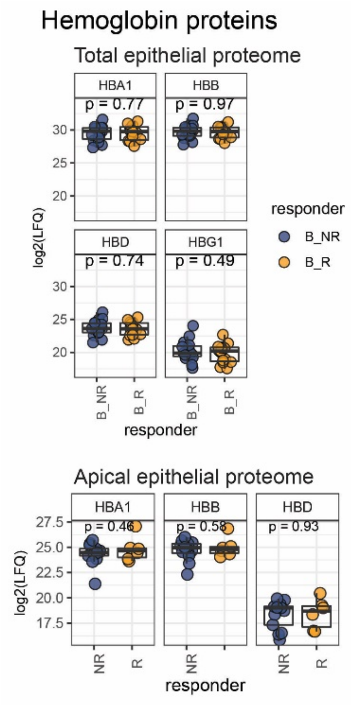

Figure S7 Hemoglobin protein presence in laser capture microdissected epithelial samples

Similar abundance of hemoglobin proteins in baseline “responder” and “non-responder” epithelial samples exclude blood vessel contamination upon laser capture microdissection collection of samples as source for differential complement protein expression.. (Comparison by Welch t-test)

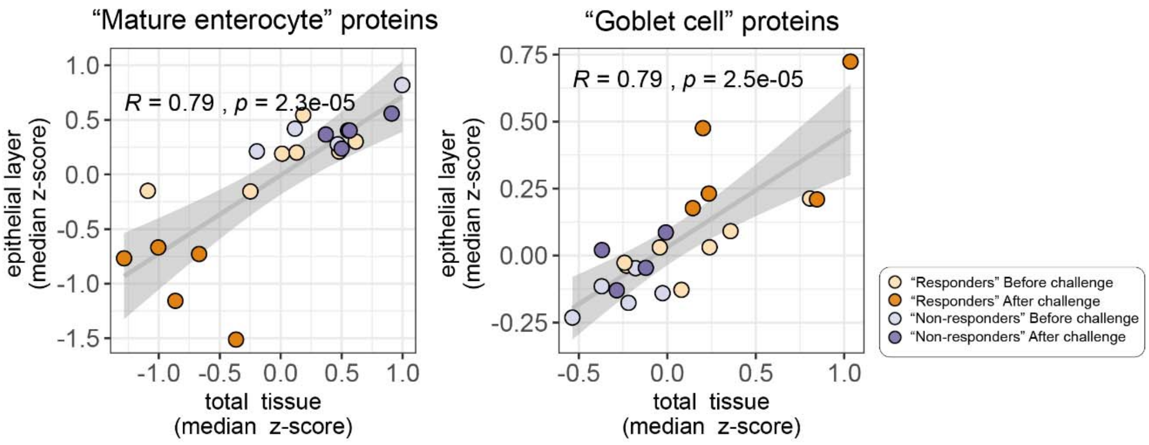

Figure S8 Comparison of cell-type specific protein expression per patient Correlation between median cell-type specific protein expression per patient biopsy from total tissue and from epithelial layer proteome datasets (Pearson correlation).

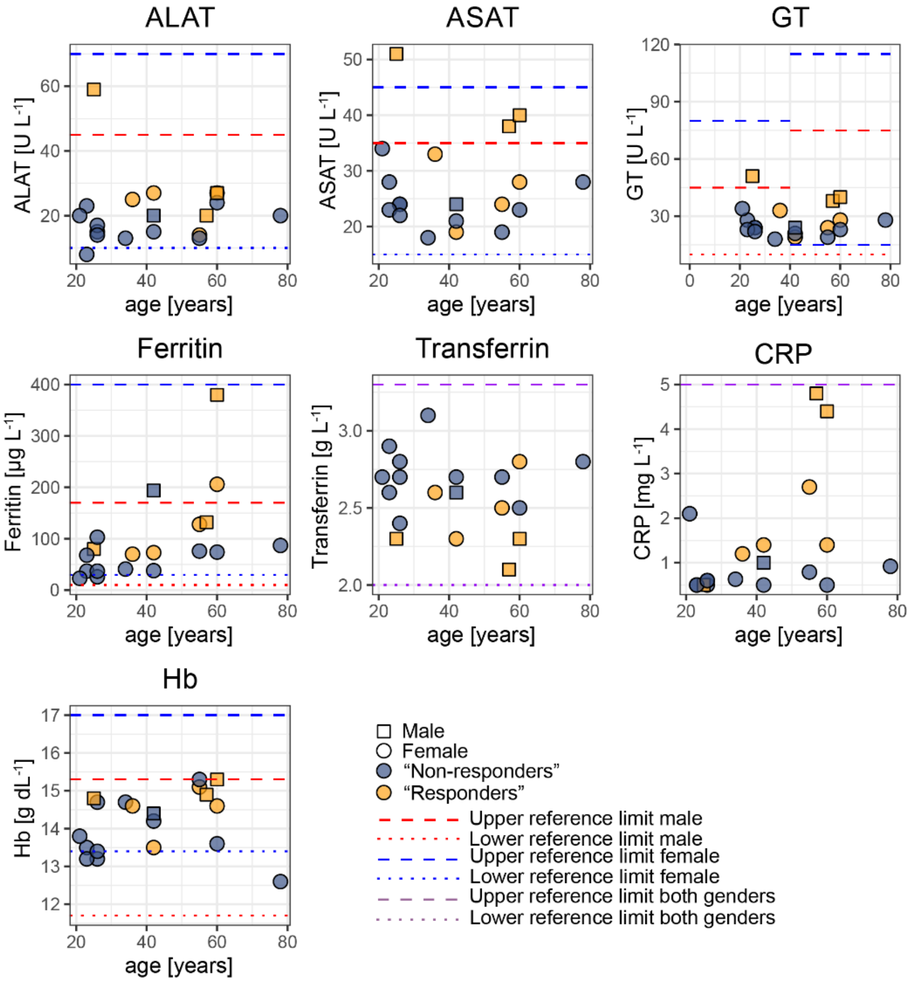

Figure S9 Biochemistry values measured at baseline before challenge as effect of age and gender Reference limits are from the local guidelines of Oslo University Hospital, Norway, of which most can be found here [12]

Table S1 Patient variables

Patient ID	CD number [a)]	Responder [b)]	Sex	HLA	GFD [months]
P1	1284	NR	F	DQ2.5	183
P2	1295	R	F	DQ2.5	165
P3	1294	NR	F	DQ8/DQ7	216
P4	1296	R	F	DQ2.5	97
P5	1299	NR	F	DQ2.5	113
P6	1298	NR	F	DQ2.5	42
P7	1300	R	F	DQ2.5	338
P8	1178	NR	F	DQ2.5/DQ8	26
P9	1302	NR	F	DQ8/8	170
P10	1303	NR	F	DQ2.5	131
P11	442	R	M	DQ2.5/DQ2.5	176
P12	1339	NR	F	DQ2.5/DQ2.2	180
P13	1340	R	M	DQ2.5/DQ2.5	34
P14	1342	NR	F	DQ2.5	60
P15	1343	R	M	DQ8/DQ2.2	239
P16	1351	NR	F	DQ2.5	32
P17	1353	R	F	DQ2.5	105
P18	1366	NR	M	DQ2.5	473
P19	1379	NR	F	DQ2.5/DQ8	147

Table S1 contd.

	Marsh score		Vh:Cd ratio			IEL/100 EC		Gluten specific $CD4^+$ T cells			
ID	base -line	day 14	base -line	day 14	diff.	base-line	day 14	blood [c)] baseline	blood day 6	gut [d)] baseline	gut day 14
P1	1	1	2.78	2.7	0,08	27.2	26.8	5.2	-	-	-
P2	0	3	2.2	1.11	1,08	22.4	45.4	49.5	n.d.	9200	17800
P3	0	0	2.47	3.07	-0,6	22.7	23.6	0.1	-	-	-
P4	0	3	2.77	1.63	1,14	21.9	48.6	1.4	-	-	-
P5	0	1	3.23	3.44	-0,21	23.9	26.5	5	55.9	6000	10800
P6	0	0	3.27	2.75	0,52	17.9	19.1	1.4	-	-	-
P7	0	3	2.21	1.43	0,78	15.8	52.9	12.1	881.4	13800	31100
P8	0	1	2.88	2.52	0,36	24.4	33.1	3.1	-	-	-
P9	0	1	2.46	2.06	0,4	21.2	37.5	3	-	-	-
P10	1	1	2.76	2.59	0,18	33.6	28.8	5.6	-	-	-
P11	3	3	1.86	1.87	-0,01	26.8	56.9	46.8	260.6	22200	22800
P12	1	1	1.97	1.84	0,13	29.7	48.4	3.3	22.9	3000	8000
P13	0	1	2.52	2.17	0,35	21.4	59.5	2.2	169.4	8200	39900
P14	0	1	2.86	2.01	0,85	16.2	72.4	1.6	21.0	1000	5000
P15	0	3	2.78	1.52	1,27	12.4	50.6	6.9	-	-	-
P16	0	1	3	2.4	0,59	31.8	50.8	2.4	-	-	-
P17	0	1	2.73	2.16	0,57	22.3	38.8	10.7	-	-	-
P18	1	0	2.91	3.19	-0,28	31.5	21.7	0	-	-	-
P19	0	1	3.69	2.53	1,16	23.4	36	6.5	-	-	-

Table S1 contd.

ID[e)]	ALAT [U L^{-1}]	ASAT [U L^{-1}]	ALP [U L^{-1}]	GT [U L^{-1}]	Transferrin [g L^{-1}]	Hb [g dL^{-1}]	Ferritin [µg L^{-1}]	CRP [d)] [mg L^{-1}]	TNF-α
P1	13	18	64	64	3.1	14.7	41	0.63	72.4
P2	27	19	62	62	2.3	13.5	73	1.4	117.25
P3	23	28	42	42	2.9	13.5	37	0.5	61.78
P4	14	24	71	71	2.5	15.1	128	2.7	124.03
P5	20	34	50	50	2.7	13.8	23	2.1	86.38
P6	15	21	48	48	2.7	14.2	38	0.5	89.84
P7	27	28	70	70	2.8	14.6	206	1.4	75.91
P8	13	19	51	51	2.7	15.3	76	0.79	93.3
P9	15	24	43	43	2.7	13.2	26	0.5	86.38
P10	20	28	72	72	2.8	12.6	87	0.92	68.87
P11	20	38	82	82	2.1	14.9	132	4.8	90.13
P12	24	23	42	42	2.5	13.6	74	0.5	65.57
P13	27	40	68	68	2.3	15.3	380	4.4	103.66
P14	17	24	32	32	2.8	14.7	37	0.6	98.62
P15	59	51	66	66	2.3	14.8	80	0.5	101.99
P16	8	23	38	38	2.6	13.2	68	0.5	61.95
P17	25	33	33	33	2.6	14.6	70	1.2	65.57
P18	20	24	53	53	2.6	14.4	194	1	86.69
P19	14	22	70	70	2.4	13.4	103	0.6	n.d.

[a)] The clinical variables have previously been published by Sarna et al. [1]; [b)] Responders are defined from tissue proteome analysis (Figure 1B); [c)] Number of HLA-DQ:gluten tetramer-binding effector-memory gut-homing CD4+ T cells per million CD4+ T cells in blood from [1] ; [d)] Number of HLA-DQ:gluten tetramer-binding CD4+ T cells per million CD4+ T cells in gut biopsies [2]; [e)] Serum biochemistry measured at baseline before gluten challenge. TNF-α was measured in plasma as part of the Bio-Plex Pro Human Cytokine 27-plex Assay as reported in [1]. [f)] CRP: LOD = 0.6. Values <0.6 are shown as 0.5 as this value was used for calculations in Figure 4 n.d.= not done

List of non-standard abbreviations

ALAT (Alanine aminotransferase), ASAT (aspartate aminotransferase), ALP (alkaline phosphatase), CeD (celiac disease), CRP (C-reactive protein), FFPE (formalin fixed paraffin embedded), GT (gamma glutamyl transferase), GOBP (Gene Ontology Biological Processes), GOCC (Gene Ontology Cellular Compartment), GOMF (Gene Ontology Molecular Function), GFD (gluten-free diet), Hb (Hemoglobin), IEL (intraepithelial lymphocyte), KEGG (Kyoto Encyclopedia of Genes and Genomes), LC (liquid chromatography), LFQ (label-free quantification), LCM (laser capture microdissection), MS (mass spectrometry), MS/MS (tandem mass spectrometry), PCA (principle component analysis), PC1 (principle component 1), TNF-α (tumor necrosis factor alpha), Vh:Cd (villus height to crypt depth),

References

[1] V K Sarna, G I Skodje, H M Reims, L F Risnes, S Dahal-Koirala, L M Sollid,K E A Lundin, *Gut.* **2018**, *67* (9), 1606-1613.

[2] L F Risnes, A Christophersen, S Dahal-Koirala, R S Neumann, G K Sandve, V K Sarna, K E Lundin, S W Qiao,L M Sollid, *J Clin Invest.* **2018**, *128* (6), 2642-2650.

[3] A E V Tutturen, S Dorum, T Clancy, H M Reims, A Christophersen, K E A Lundin, L M Sollid, G A de Souza,J Stamnaes, *Am J Pathol.* **2018**, *188* (7), 1563-1579.

[4] J A Vizcaino, A Csordas, N del-Toro, J A Dianes, J Griss, I Lavidas, G Mayer, Y Perez-Riverol, F Reisinger, T Ternent, Q W Xu, R Wang,H Hermjakob, *Nucleic Acids Res.* **2016**, *44* (D1), D447-56.

[5] J Cox,M Mann, *Nat Biotechnol.* **2008**, *26* (12), 1367-72.

[6] J Cox, N Neuhauser, A Michalski, R A Scheltema, J V Olsen,M Mann, *J Proteome Res.* **2011**, *10* (4), 1794-805.

[7] J Cox, M Y Hein, C A Luber, I Paron, N Nagaraj,M Mann, *Mol Cell Proteomics.* **2014**, *13* (9), 2513-26.

[8] S Tyanova,J Cox, *Methods Mol Biol.* **2018**, *1711*, 133-148.

[9] J Cox,M Mann, *BMC Bioinformatics.* **2012**, *13 Suppl 16*, S12.

[10] Y M Nusse, A K Savage, P Marangoni, A K M Rosendahl-Huber, T A Landman, F J de Sauvage, R M Locksley,O D Klein, *Nature.* **2018**, *559* (7712), 109-113.

[11] S Tyanova, T Temu, P Sinitcyn, A Carlson, M Y Hein, T Geiger, M Mann,J Cox, *Nat Methods.* **2016**, *13* (9), 731-40.

[12] P Rustad, P Felding, L Franzson, V Kairisto, A Lahti, A Martensson, P Hyltoft Petersen, P Simonsson, H Steensland,A Uldall, *Scand J Clin Lab Invest.* **2004**, *64* (4), 271-84.
